# CDH3 Is an Effective Serum Biomarker of Colorectal Cancer Distant Metastasis Patients

**DOI:** 10.7150/jca.98337

**Published:** 2024-08-13

**Authors:** Jiayin Song, Yu Jin, Shuoqing Fan, Yue Wei, Dong Dong, Li Jia, Shuxuan Fan, Aimin Zhang, Wei Zhou, Wenna Jiang, Li Ren

**Affiliations:** 1 Department of Clinical Laboratory, Tianjin Medical University Cancer Institute and Hospital, National Clinical Research Center for Cancer, Tianjin's Clinical Research Center for Cancer, Tianjin Key Laboratory of Digestive Cancer, Tianjin 300060, China.; 2 Clinical medical college of Tianjin Medical University, Tianjin, China.; 3 Department of Radiology, Tianjin Medical University Cancer Institute & Hospital, National Clinical Research Center for Cancer, Tianjin's Clinical Research Center for Cancer, Tianjin Key Laboratory of Digestive Cancer, Tianjin, China.

**Keywords:** colorectal cancer, CDH3, biomarkers, palliative chemotherapy

## Abstract

Few robust biomarkers are available for distant metastatic colorectal cancer (CRC) patients. Aberrant high expression of CDH3 has been reported in advanced CRC patients, but the value of CDH3 as a biomarker for the diagnosis and prognosis of distant metastatic CRC patients remains to be evaluated. In this study, we explored the serum levels of CDH3 in different stages of CRC patients and sought to determine whether serum CDH3 serves as an independent biomarker for distant metastatic CRC patients. We analyzed the serum CDH3 levels by ELISA in a cohort of CRCs (n=96) and normal controls (n=28). We compared the serum CDH3 levels between normal controls and different stages of CRCs. As a potential diagnostic marker of distant metastatic CRC, the specificity and sensitivity of serum CDH3 were evaluated. Multivariate analysis was also performed to determine whether serum CDH3 was an independent risk factor. Moreover, the changes of serum CDH3 levels were monitored and analyzed before and after palliative chemotherapy. Serum levels of CDH3, CA24-2, CA19-9, CA72-4, and CEA were significantly elevated in distant metastatic CRCs. CA24-2 (*r*=0.24, *P*=0.01), CA19-9 (*r*=0.20, *P*=0.03), CA72-4 (*r*=0.64, *P*<0.0001), and CEA (*r*=0.31, *P*=0.0012) all had a certain correlation with CDH3. After three cycles of palliative chemotherapy, levels of CDH3, CA24-2, CA19-9, CA72-4, and CEA of partial response CRCs were reduced to 38.8% (95% confidence interval [CI]: 30.95%-53.77%), 57.73% (95% CI: 2.085%-73.83%), 50.33% (95% CI: 9.935%-79.42%), 74.74% (95% CI: 25.21%-88.00%), and 59.16% (95% CI: 12.65%-83.56%) of baseline, respectively. The areas under the receiver operating characteristic curves of CDH3, CA24-2, CA19-9, CA72-4, and CEA with chemotherapy response were 0.900, 0.597, 0.635, 0.608, and 0.507, respectively. Serum CDH3 is an effective serum biomarker for the diagnosis of distant metastatic CRCs and monitoring response to palliative chemotherapy in distant metastatic CRCs.

## Introduction

Colorectal cancer (CRC) is the third most common cancer type and the second leading cause of cancer-related mortality worldwide 1. An aging population, poor diet, and unhealthy lifestyle are all associated with increased risk of CRC. High recurrence and metastasis rates are the main reasons for the high mortality rate of CRC. Studies have found that approximately 50% of primary CRCs eventually develop metastatic disease, with 25% being synchronous metastases and another 25% developing metastases throughout the disease course 2,3. According to current recommendations, CRCs without distant metastasis can be treated with multiple therapies, resulting in a good 5-year survival rate. Given the lack of surgical intervention, CRCs with distant metastasis have a poor prognosis, with a 5-year survival rate of less than 10% 2,3. Unfortunately, the underlying mechanisms of CRC metastasis have not been completely elucidated, and there is a lack of clinical biomarkers for the diagnosis of colorectal cancer distant metastasis and the monitoring of chemotherapy efficacy.

A key mechanism by which cancer cells strengthen their ability to invade and metastasize is the dissolution of intercellular adhesions and the acquisition of a more aggressive mesenchymal phenotype as part of epithelial-mesenchymal transition 4,5. Cadherins are a family of transmembrane glycoproteins that mediate calcium-dependent cell adhesion and have important functions in maintaining normal tissue structure. P-Cadherin (CDH3) is one of the lesser-known cadherins. CDH3 is encoded by the *CDH3* gene in humans, initially discovered in the placenta, and involved in embryonic development 6. CDH3 plays an important role in the regulation of cell differentiation, shape, polarity, growth, and migration 7,8. Moreover, the association between abnormal expression of CDH3 and cancer prognosis has been reported in many cancers, such as pancreatic cancer 9, thyroid cancer 10, tongue squamous cell carcinoma 11-13, liver cancer 14, gastric cancer 15, esophageal cancer 16,17, cholangiocarcinoma 18, renal cell carcinoma 19, breast cancer 20-22, prostate cancer 23, lung cancer 24 and glioblastoma 25. Besides, *CDH3* has been identified as a susceptibility gene for CRC 26. Demethylation of the *CDH3* gene locus and subsequent upregulation of its expression are frequently detected in advanced CRC 27,28. Interestingly, aberrant high expression of CDH3 is associated with good prognosis in colon adenocarcinoma 29. The research by Sharma G emphasizes the crucial role of CDH3 in regulating cell proliferation, migration, and apoptosis in colorectal cancer 30. However, limited effort has been devoted to defining the prognostic value of CDH3 as a biomarker for CRC, especially metastatic CRC.

In our previous study, we analyzed the Oncomine and TCGA databases and discovered a significant increase in the level of CDH3 in CRC tissues compared to normal tissues. In this study, we first explored the serum levels of CDH3 in different stages of CRCs. Using well known biomarkers (CA24-2, CA19-9, CA72-4, and CEA) of CRC as benchmarks, serum CDH3 demonstrated superior performance than CA24-2, CA19-9, and CEA for distant metastatic CRCs. Our results proposed serum CDH3 as an effective serum biomarker for the diagnosis of distant metastatic CRCs and monitoring response to palliative chemotherapy in distant metastatic CRCs.

## Materials and Methods

### Studied Population

A total of 124 subjects were enrolled in this study, including 96 CRCs and 28 normal controls (NCs). CRCs were diagnosed at the Tianjin Medical University Cancer Institute and Hospital (Tianjin, China) between August 2020 and December 2020. The inclusion criteria were as follows: (1) patients who met the diagnostic criteria for CRC and confirmed by pathological examination, (2) CRCs did not undergo chemotherapy or radiotherapy, and (3) clinical and pathological data of CRCs were complete. The exclusion criteria were as follows: (1) CRCs with hypertension, heart disease, diabetes, glaucoma, or other underlying diseases; (2) patients with other tumors; (3) CRCs with intellectual disability or other serious mental illness; and (4) CRCs with liver, kidney, or other gastrointestinal diseases. The NCs were individuals who underwent physical examination at Tianjin Medical University Cancer Institute and Hospital (Tianjin, China) from August to December 2020. The inclusion criteria were people aged ≥ 18 years, and the exclusion criteria were the same as described above. The clinical features of the study subjects (28 NCs and 96 CRCs) were shown in **Table 1**.

All procedures involving human participants in this study complied with the Declaration of Helsinki (2013 revision). The study was approved by the Tianjin Medical University Cancer Institute and hospital ethics committee (bc2021223), and informed consent was obtained from all patients.

### Blood Sample Collection and Storage

The subjects were required to fast overnight (minimum of 8 h), and venous blood samples were collected from 7 AM to 8 AM the following morning using a 5 ml ethylenediamine tetraacetic acid vacutainer and centrifuged at 3,000 × g for 15 min at 4 °C within 2 h. The supernatant (serum) was split into two tubes and immediately stored frozen at -80 °C until use.

### Serum CDH3 Assays

The serum levels of CDH3 were determined via enzyme-linked immunosorbent assay (ELISA) according to the manufacturer's protocol, in duplicate samples, using a human CDH3 ELISA duo set kit (R&D Systems, Minneapolis, MN, USA) and microplate reader (Thermo Scientific™ Multiskan™ FC, USA).

### Database Analysis

In the Oncomine database (www.oncomine.org), we entered the cancer type “CRC” and chose the differential gene analysis module (cancer vs. normal analysis) to retrieve the results. In this study, a *P*-value < 0.01, a log_2_(Fold Change) of 5, and a gene rank in the top 10% were set as the significance thresholds.

The data of CDH3 expression levels of CRC were downloaded from The Cancer Genome Atlas data portal (TCGA). The gene *CDH3* expression units of the TCGA-COAD datasets were log2[FPKM] + 1.

The Human Protein Atlas (HPA, https://www.proteinatlas.org) is a publicly available database containing antibody-based localization data for human proteins. We analyzed CDH3 protein expression in CRC via immunohistochemistry (IHC) analysis.

### Evaluation of the Efficacy of Palliative Chemotherapy in distant metastatic CRCs

We thoroughly tracked the course of three cycles of palliative chemotherapy in 24 distant metastatic CRCs. For distant metastatic CRCs enhanced computed tomography (CT) scan was performed to detect tumor size prior to the first chemotherapy treatment. CA24-2, CA19-9, CA72-4, CEA, and CDH3 were detected after the end of a chemotherapy cycle and prior to the next chemotherapy cycle. At the end of three cycles of chemotherapy, enhanced CT scan was performed on stage IV CRCs again, and the specialists assessed the response to chemotherapy based on Response Evaluation Criteria In Solid Tumours (RECIST) v1.1 31. Complete response (CR) was defined as the disappearance of all tumor lesions. Partial response (PR) was a reduction in total tumor size of > 30%. Stable disease (SD) was a reduction of < 30% or a growth of < 20%. Progressive disease (PD) was a growth of > 20% or occurrence of new lesions. All changes were relative to baseline imaging.

### Statistical Analysis

Statistical analyses were performed using SPSS software (version 22.0; IBM Corp, Armonk, NY, USA). Continuous data were tested for normality and reported as mean and standard deviation (SD) or median (25th-75th percentiles). Differences between groups were assessed for significance using Student's *t*-test or Wilcoxon rank-sum test for continuous variables, as appropriate. The enumeration data were expressed as n (%).

## Results

### CDH3 was significantly elevated in distant metastatic CRCs

**Figure 1:**
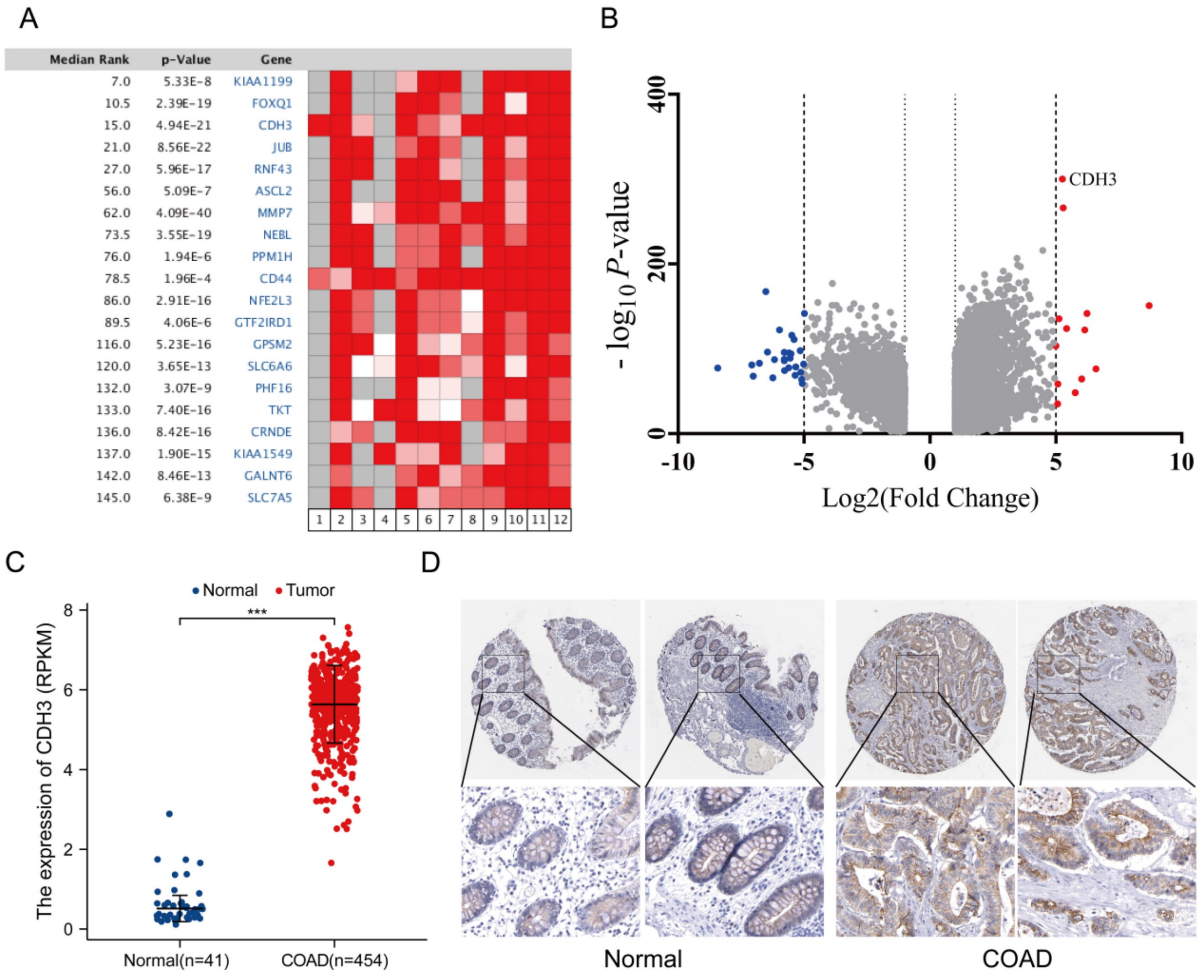
** Database analysis.** (A) Heatmap of differential gene expression in CRC and normal tissue in the Oncomine database. (B) Volcano plot of the top 100 differentially expressed genes in CRC and normal tissue in the Oncomine database. (C) Relative CDH3 expression in TCGA including 454 CRC samples and 41 normal tissues (unpaired *t*-test, *P*<0.0001). (D) Representative immunochemistry staining of CDH3 in COAD and normal tissues from the HPA database (magnification unavailable). (* *P* < 0.05, ** *P* < 0.01, *** *P* < 0.001, ****P < 0.0001).

Analysis of the Oncomine and TCGA databases indicated that the level of CDH3 was significantly increased in CRC tissues compared with that in normal tissues, as evidenced by expression profiling (**Figures 1A-1C**). In agreement with this, IHC staining demonstrated higher expression of CDH3, as indicated by brownish yellow staining, in normal tissue than tumor tissue (**Figure 1D**).

**Figure 2:**
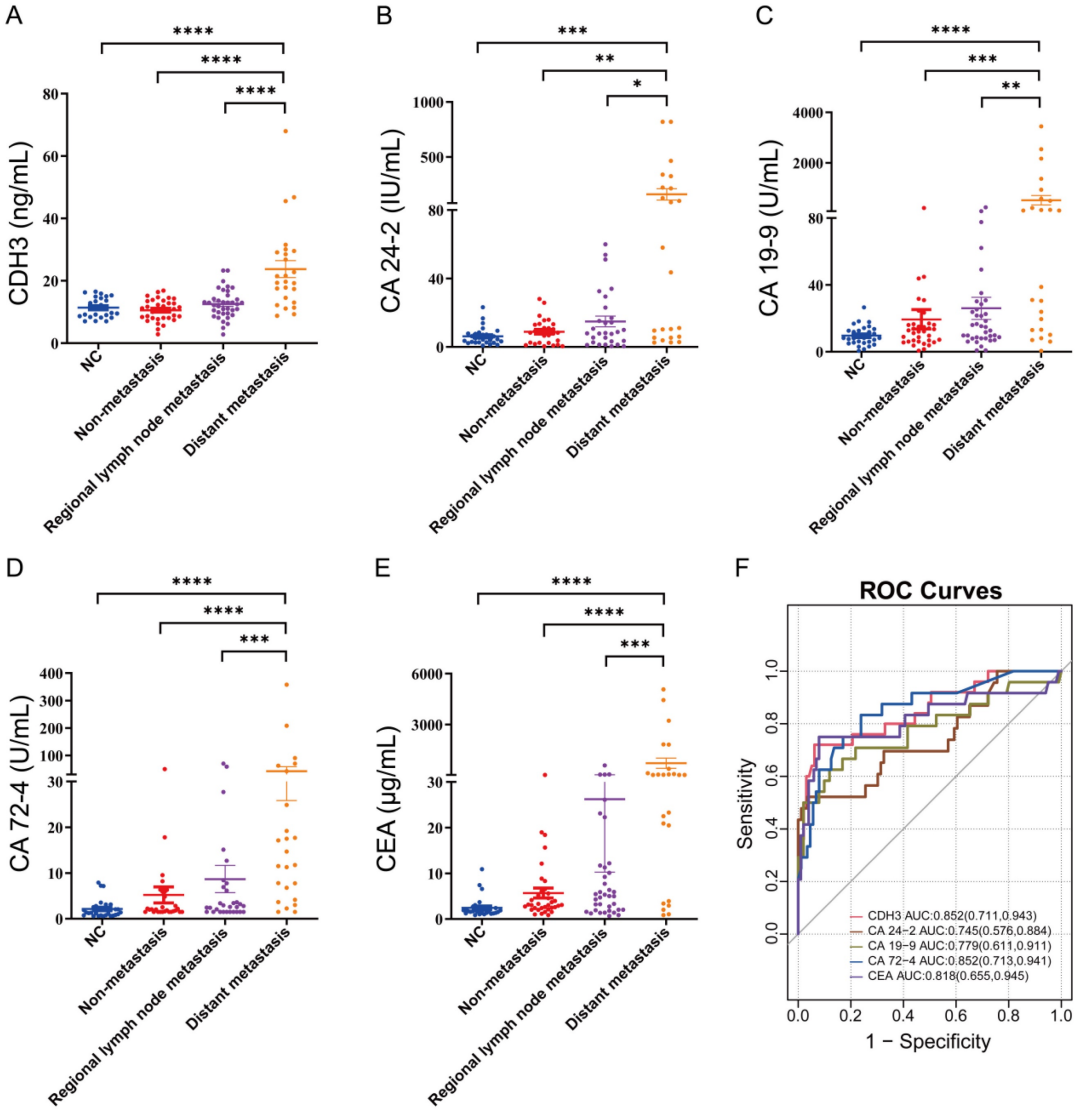
** Expression of CDH3, CA24-2, CA19-9, CA72-4, and CEA in NCs and CRCs.** (* *P* < 0.05, ** *P* < 0.01, *** *P* < 0.001, ****P < 0.0001).

According to the tumor metastatic status, CRCs were divided into non-metastatic CRCs (n=35), regional lymph node metastatic CRCs (n=37), and distant metastatic CRCs (n=24). Systemic chemotherapy regimens of capecitabine + oxaliplatin (XELOX) or the combination of XELOX + bevacizumab were the mainstay of treatment for stage IV CRCs. To further gain insight into the association between CDH3 expression and prognosis of CRC, we explored the distribution of CDH3 in the serum of NCs versus different stages of CRCs. Surprisingly, no significant difference in serum CDH3 distribution was found between NCs and non-metastatic CRCs or regional lymph node metastatic CRCs. Notably, the level of serum CDH3 in distant metastatic CRCs was significantly higher compared to NCs (**Figure 2A**), pointing to serum CDH3 as a potential biomarker for distant metastatic CRCs.

### CDH3 can be used as a serum biomarker of distant metastatic CRCs

**Figure 3:**
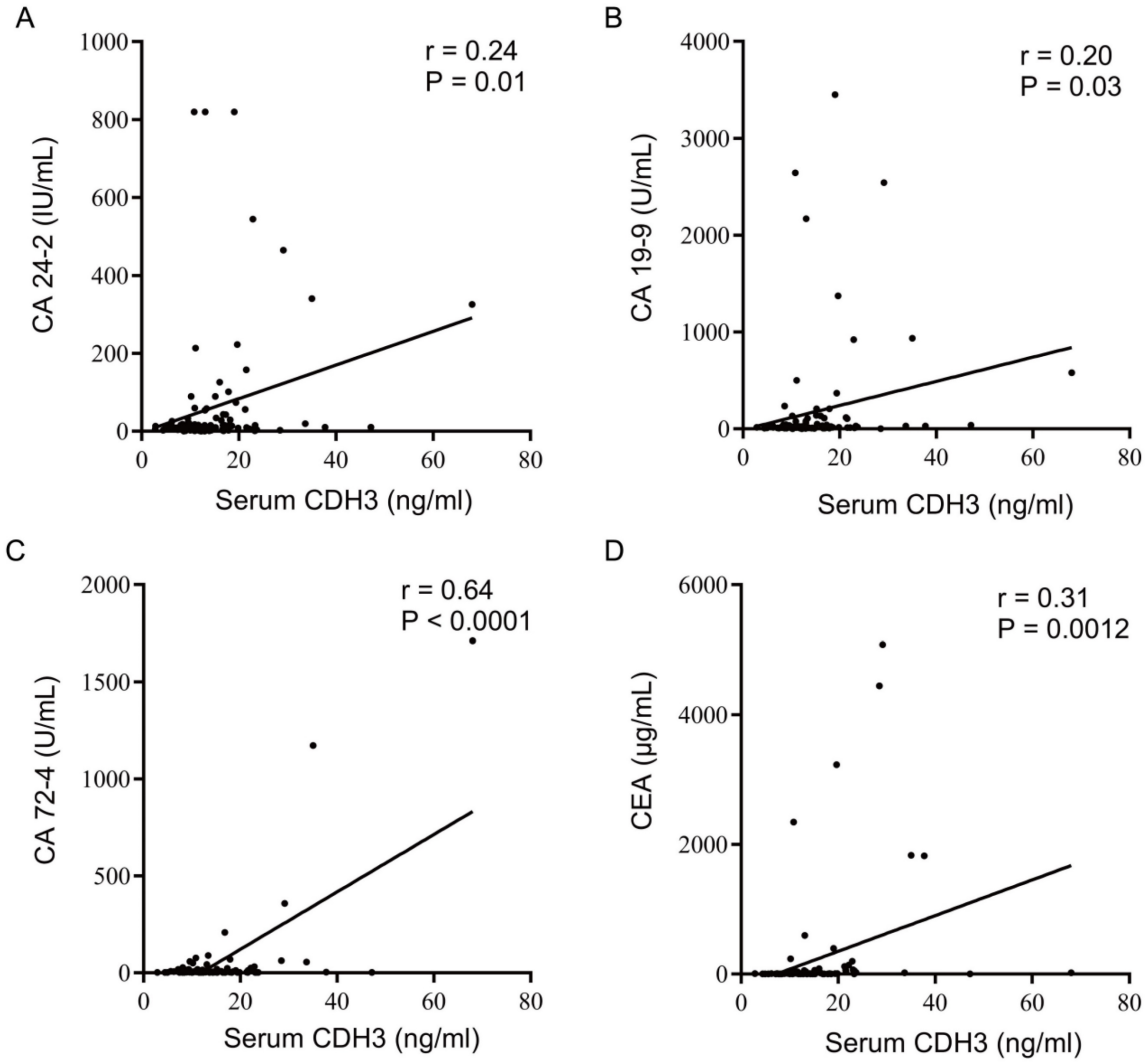
Correlation analysis of CDH3, CA24-2, CA19-9, CA72-4, and CEA.

Given its high serum levels in distant metastatic CRC, we moved forward to benchmark the performance of serum CDH3 against four existing serum biomarkers of CRC. We assessed the serum levels of carbohydrate antigen 24-2 (CA 24-2), carbohydrate antigen 19-9 (CA 19-9), carbohydrate antigen 72-4 (CA 72-4), and carcinoembryonic antigen (CEA) in different stages of CRCs. As expected, CA 24-2, CA 19-9, CA 72-4, and CEA were significantly elevated in distant metastatic CRCs (**Figures 2B-2E**). In addition, the area under the receiver operating characteristic curves (AUCs) was 0.852 for both CDH3 and CA 72-4 in the diagnosis of distal metastatic CRCs, superior over CA 24-2 (0.745), CA 19-9 (0.779), and CEA (0.818) (**Figures 2F**). According to the maximum principle of Jordan's index, when the concentration of serum CDH3 was 17.245 ng/ml, the sensitivity was 0.72, and the specificity was 0.938. The sensitivities of CA 24-2, CA 19-9, CA 72-4, and CEA were 0.522, 0.625, 0.833, and 0.75 respectively, while the specificities were 0.965, 0.881, 0.761 and 0.921. Interestingly, CDH3 showed a strong correlation (*r*=0.64, *P*<0.0001) with CA 72-4, and to a lesser extent, with other markers (**Figure 3**). To further assess whether serum CDH3 is an independent factor, we performed binary and multivariate logistic regression analyses. Age and gender were not associated with the occurrence of distant metastasis of CRC (*P*>0.05). In contrast, CDH3 and the tumor markers CA 24-2, CA 19-9, CA 72-4, and CEA (*P*<0.001) were associated with the occurrence of distant metastasis of CRCs (**Table 2**). Multivariate logistic regression analysis revealed that elevated serum levels of CDH3, CA19-9, and CA724 were identified as independent risk factors for distant metastasis of CRCs, with odds ratios of 34.852, 12.298, and 1.039 respectively (**Table 3**). The accuracy rates of CDH3, CA 24-2, CA 19-9, CA 72-4, and CEA were as follows: 95.16% (118/124), 80.64% (100/124), 81.45% (101/124), 80.64% (100/124), and 68.55% (85/124) respectively (**Table 2**). This indicates the strong predictive power of high CDH3 expression in predicting distant metastasis in colorectal cancer. Therefore, CDH3 shows the potential to be a serum biomarker of distant metastatic CRCs.

### Decreased serum CDH3 levels were consistent with the PR of chemotherapy response

**Figure 4:**
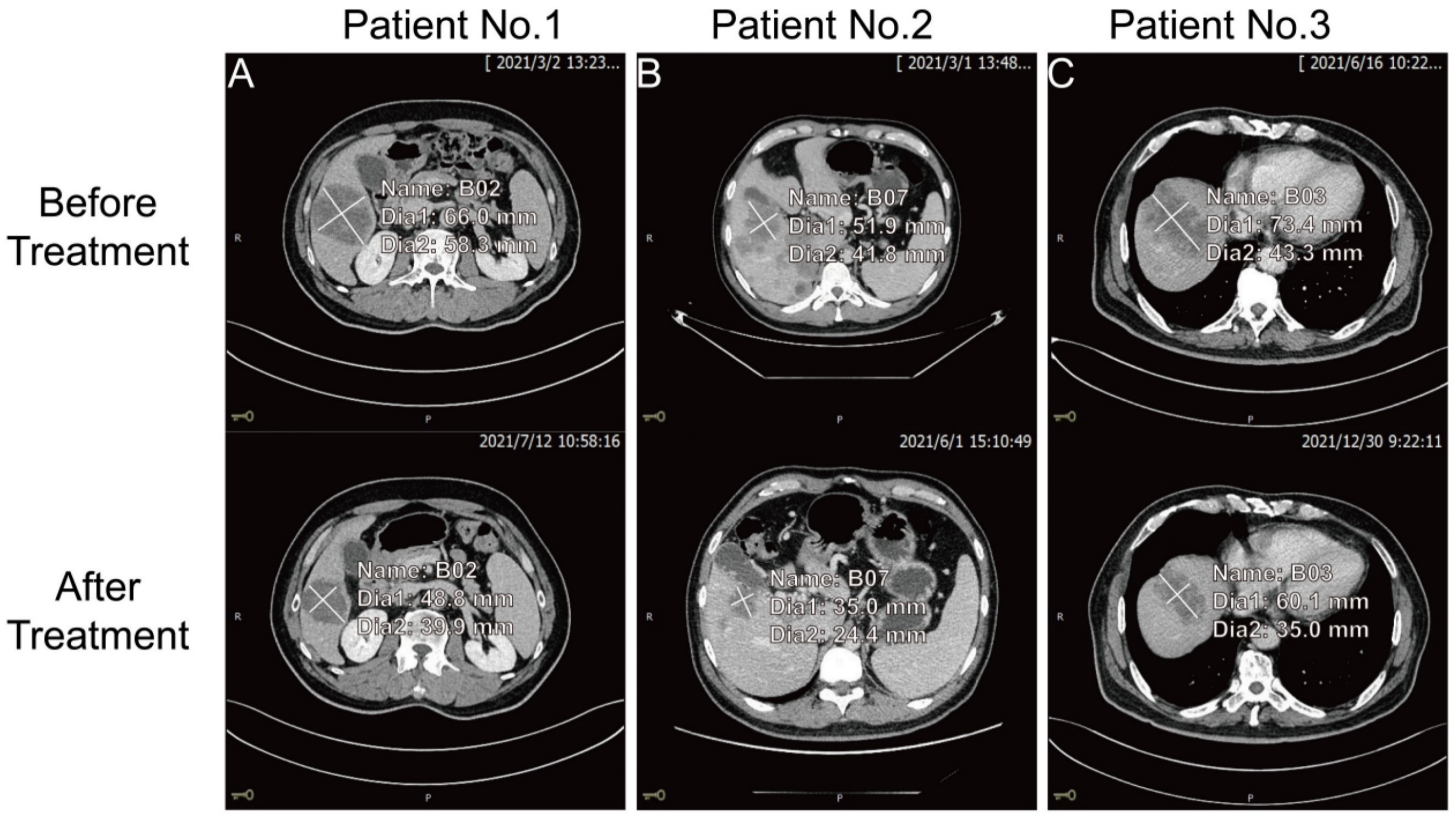
The comparison of tumor size at the site of liver metastasis from colorectal cancer before and after three chemotherapy treatments for three patients in the PR group.

**Figure 5:**
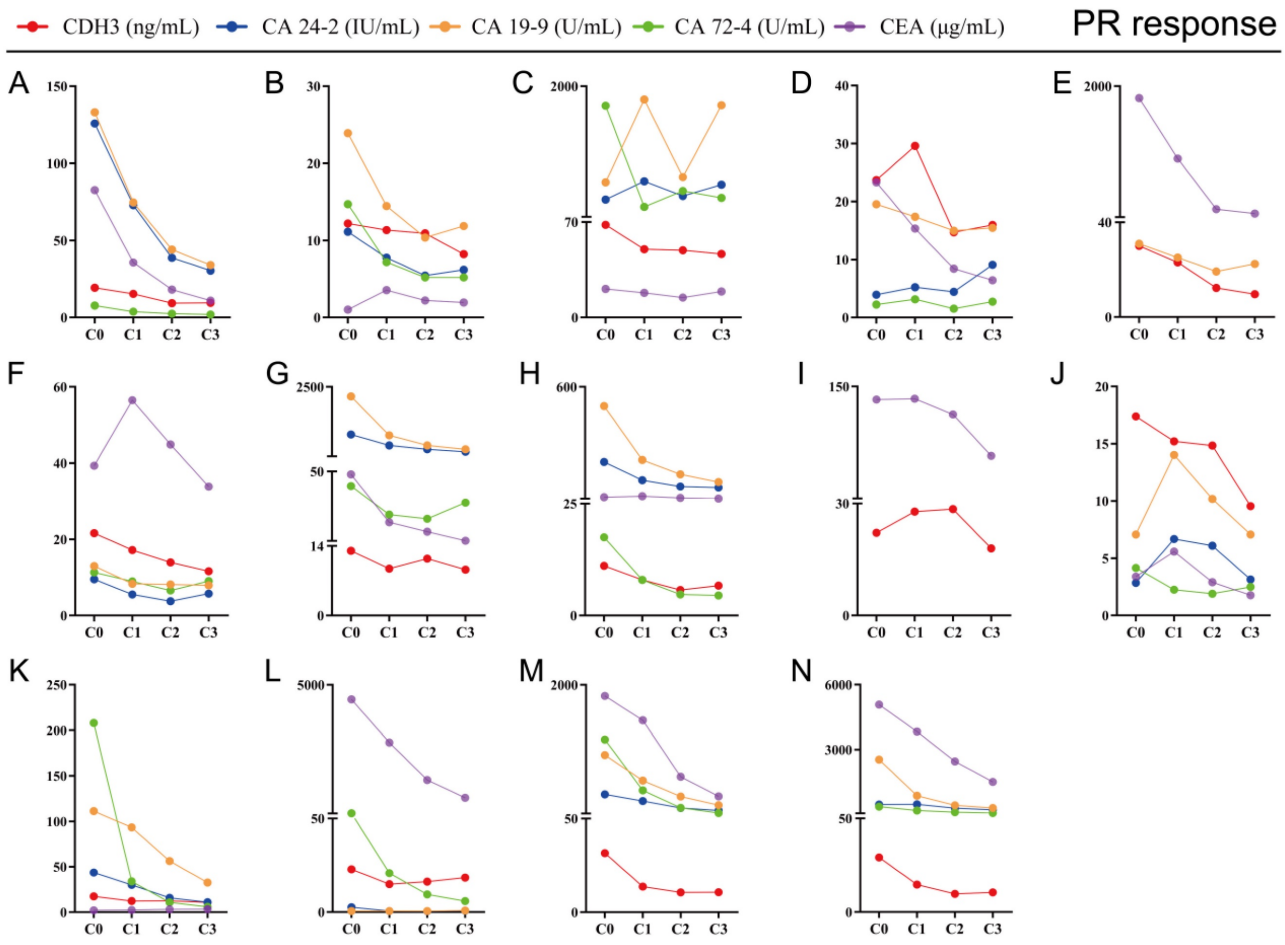
** Tracking data of CDH3, CA24-2, CA19-9, CA72-4, and CEA in 14 CRCs with PR response to chemotherapy.** C0, received no chemotherapy; C1, received one cycle of chemotherapy treatment; C2, received two cycles of chemotherapy treatments; C3, received three cycles of chemotherapy treatments.

**Figure 6:**
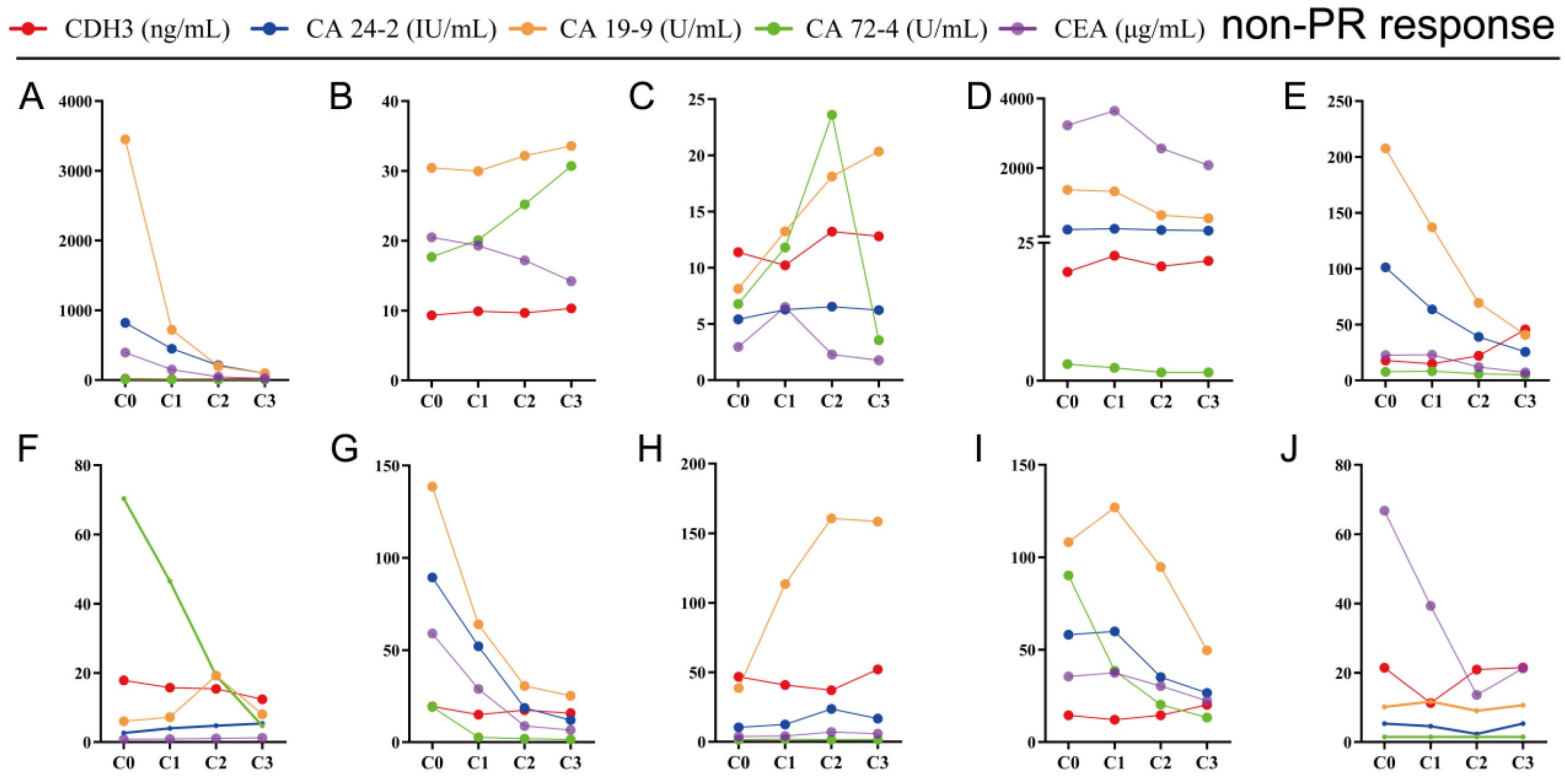
** Tracking data of CDH3, CA24-2, CA19-9, CA72-4, and CEA in 10 CRCs with non-PR response to chemotherapy.** C0, received no chemotherapy; C1, received one chemotherapy treatment; C2, received two chemotherapy treatments; C3, received three chemotherapy treatments.

The key to making a correct treatment plan is to monitor and judge the efficacy of chemotherapy in patients with distant metastatic CRCs. By evaluating the treatment response, 14 distant metastatic CRCs reached PR, and the remaining CRCs were classified as non-PR (including CRCs evaluated as PD or SD on chemotherapy). Apparent decrease of tumor size was observed at the site of liver metastasis from colorectal cancer (n=3) after three chemotherapy treatments in the PR group (**Figure 4**). Our follow-up test found that the serum CDH3 level in the PR group gradually decreased with the increase of numbers of chemotherapy sessions, which was not observed in non-PR. CA 24-2, CA 19-9, CA 72-4, and CEA in the PR group also showed a decrease in overall chemotherapy, while strong fluctuations were noted in the levels of these biomarkers in the non-PR group (**Figures 5** and **6**). However, no significant differences were observed in the serum levels of these markers between PR group and non-PR group after chemotherapy, except for CDH3 (**Table 4**).

**Figure 7:**
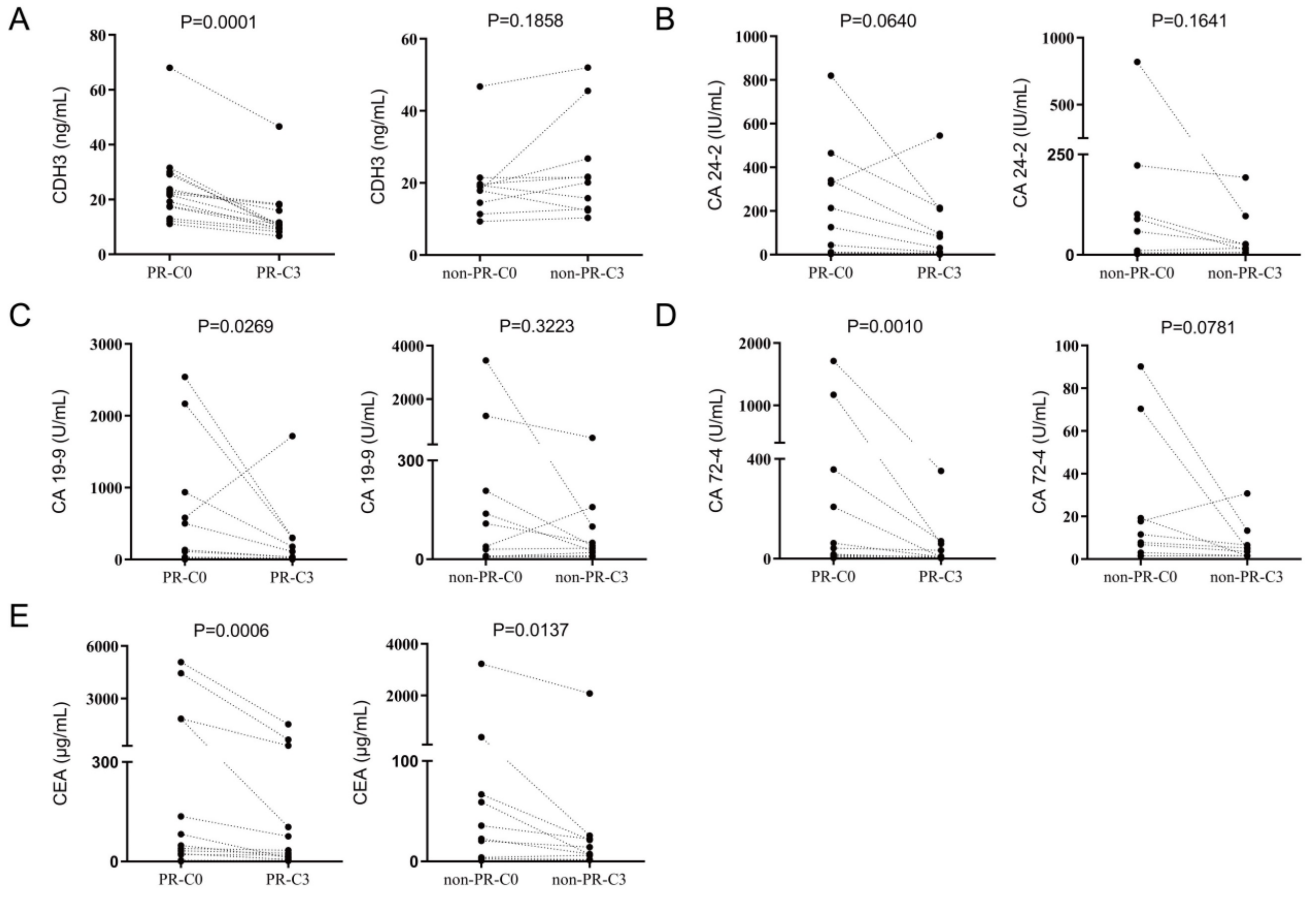
** Comparison of CDH3, CA24-2, CA19-9, CA72-4 and CEA before and after three chemotherapy treatments in PR group and non-PR group.** C0, received no chemotherapy; C3, received three cycles of chemotherapy.

We then focused on patients receiving three cycles of chemotherapy treatments and compared the serum levels of CDH3, CA 24-2, CA 19-9, CA 72-4, and CEA before and after three cycles of chemotherapy treatments. Serum levels of CDH3, CA 19-9, CA 72-4, and CEA were all reduced in the PR group after three cycles of chemotherapy except for CA 24-2 (**Figure 7**). In the non-PR group, serum levels of CDH3, CA 24-2, CA 72-4, and CA 19-9 showed no significant difference before and after three cycles of chemotherapy, whereas CEA decreases significantly in the non-PR group (**Figure 7**).

**Figure 8:**
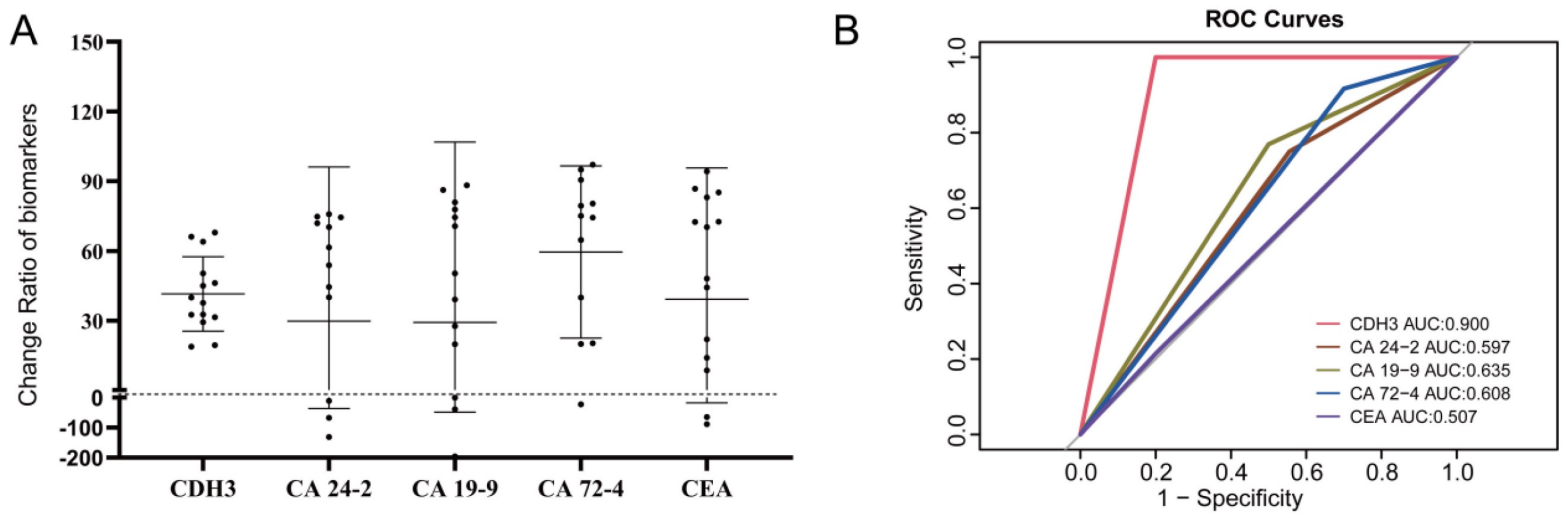
** Reduction in CDH3, CA24-2, CA19-9, CA72-4, and CEA in the PR group after three chemotherapy treatments was consistent with chemotherapy response.** (A) Changes in CDH3, CA24-2, CA19-9, CA72-4, and CEA before and after three chemotherapy treatments in the PR group. (B) The consistency of CDH3, CA24-2, CA19-9, CA72-4, and CEA response to chemotherapy.

The reduction degrees of CA24-2, CA724, CA19-9, CEA, and CDH3 before and after three cycles of chemotherapy were analyzed in the PR group. After three cycles of palliative chemotherapy, CDH3, CA24-2, CA19-9, CA72-4, and CEA of CRCs in the PR group were reduced to 38.8% (95% confidence interval [CI]: 30.95%-53.77%), 57.73% (95% CI: 2.085%-73.83%), 50.33% (95% CI: 9.935%-79.42%), 74.74% (95% CI: 25.21%-88.00%), and 59.16% (95% CI: 12.65%-83.56%) of the baseline, respectively (**Figure 8A**). We defined C3/C0<1 in the PR group or C3/C0≥ 1 in the non-PR group as consistent with the chemotherapy response. **Table 5** showed that C3/C0<1 for CDH3 can be an important criterion for achieving PR with three cycles of palliative chemotherapy in distant metastatic CRCs. The areas under the receiver operating characteristic curves (AUCs) of CDH3 response to palliative chemotherapy was 0.900, and those of CA 24-2, CA 19-9, CA 72-4, and CEA were 0.597, 0.635, 0.608, and 0.507, respectively (**Figure 8B**). Collectively, CDH3 as a promising serum biomarker for monitoring the response to chemotherapy in distant metastatic CRCs was superior to CA 24-2, CA 19-9, CA 72-4, and CEA.

## Discussion

Palliative chemotherapy is the mainstay of treatment for distant metastatic CRCs due to the lack of surgical conditions for radical cure. The purpose of palliative chemotherapy is to extend overall survival, improve disease symptoms, and maintain quality of life as long as possible 32. Chemotherapeutic drugs are cell-killing drugs that are “indiscriminating between enemies and friends.” While acting on tumor cells, they also harm normal cells, especially those cells rapidly growing, such as hair follicle cells, bone marrow hematopoietic cells, and gastrointestinal mucosa cells. The side effects of the combined chemotherapy regime are severe, including bone marrow suppression (leukopenia and red blood cell reduction) and gastrointestinal reactions (nausea, vomiting, poor appetite, diarrhea, and constipation) 33. Oxaliplatin will have neurotoxic reactions, mainly manifested as numbness of the extremities, pain, and numbness around the lips 34. In addition, about 54% of patients were unable to work during therapy. In 74% of patients, chemotherapy caused severe or moderate impairment of daily activities and negatively affected their financial situations 35. Clinical decision-making for palliative chemotherapy in distant metastatic CRCs is sophisticated because of multiple options and multidisciplinary approaches. The effective way to ascertain the efficacy of chemotherapy is to observe the changes in tumor size by enhanced CT, but it cannot be executed frequently, considering the risk of radiation exposure and high economic and time costs. Therefore, convenient, economical, and minimally harmful biomarkers for chemotherapy monitoring are urgently needed.

The analysis of the Oncomine and TCGA databases demonstrated that CDH3 levels in CRC tissues were significantly increased compared with those in normal tissues. CDH3 emerges as an expected serum biomarker for CRC. In this study, no significant difference was found in the serum CDH3 levels between NCs, non-metastatic CRCs and regional lymph node metastatic CRCs, but the serum CDH3 level of distant metastatic CRCs significantly increased. This result indicated that CDH3 could be a unique serum biomarker for distant metastatic CRCs. CA 24-2, CA 19-9, CA 72-4, and CEA are common diagnostic and prognostic serum tumor markers for CRCs but lack specificity for CRCs 36-40. CA 24-2, CA 19-9, CA 72-4, and CEA were significantly increased in distant metastatic CRCs. We verified that CA24-2 (*r*=0.24, *P*=0.01), CA19-9 (*r*=0.20, *P*=0.03), CA72-4 (*r*=0.64, *P*<0.0001), and CEA (*r*=0.31, *P*=0.0012) were associated with CDH3. Statistically significant differences in CA72-4 values were observed between early and late-stage diseases (*P* < 0.001), as well as between patients with and without distant metastases (*P* < 0.001). This indicates a strong association between elevated CA72-4 levels and advanced disease status, highlighting its potential as a biomarker for disease progression and metastatic spread in colorectal cancer 41. CA72-4, a glycoprotein, is overexpressed in various cancers, including CRC, and serves as a diagnostic and prognostic marker 41. The strong association between CDH3 and CA72-4 implies that alterations in cell adhesion may contribute to the upregulation of CA72-4, potentially facilitating tumor spread.

The patients with distant metastatic CRCs included in this study were all treated with palliative chemotherapy, with good compliance and complete clinical data. During our assessment of distant metastatic CRCs, we found that CDH3 gradually declined in the PR group after three chemotherapy treatments, which was not reflected in the non-PR group. The numerical changes in CA 24-2, CA 19-9, CA 72-4, and CEA were also studied. CA242, CA72-4, CA19-9, and CEA showed a decrease in the PR group. Different from CA72-4 and CA19-9, CEA still exhibited a significant decrease in the non-PR group. P Byström et al. evaluated the changes in tumor markers such as CA 24-2, CA 19-9, CA 72-4, and CEA during palliative chemotherapy in advanced upper gastrointestinal adenocarcinoma (UGIA). Their research showed that baseline tumor marker levels provide prognostic information for patients with UGIA receiving palliative chemotherapy, but early tumor marker changes often fail to provide accurate information on tumor response and survival 42. CEA and CA 19-9 have been verified to exhibit synchrony with chemotherapy response and can be used as biomarkers for palliative chemotherapy monitoring in advanced gastric cancer 43,44. An increase in CEA or CA 19-9 is only conditionally appropriate for recording progression. A progression can be excluded with declining levels with high diagnostic accuracy, in which CEA offers a greater degree of certainty than CA 19-9 45. CA72-4 was a statistically significant independent risk factor for the prognosis of CRC patients 46. The above findings were consistent with our findings.

The aim of this study was to find an indicator that would respond to the effect of palliative chemotherapy. The expression of CDH3, CA 24-2, CA 19-9, CA 72-4, and CEA after three times treatments in CRC patients with distant metastases weren't associated with the response to chemotherapy (**Table 4**). Measurements of serum tumor markers CA 24-2, CA 19-9, CA 72-4, and CEA were of interest during palliative chemotherapy for distant metastatic CRCs. The ratio of CDH3 after the third chemotherapy to the initial CDH3 (C3/C0) was significant for the response to chemotherapy. (*P*<0.05, **Table 5**). The AUCs of CA24-2, CA19-9, CA72-4, and CEA (C3/C0) in response to palliative chemotherapy in distant metastatic CRCs were 0.597, 0.635, 0.608, and 0.507, respectively. CA24-2, CA19-9, CA72-4, and CEA of CRCs in the PR group were reduced to 57.73% (95% CI: 2.085%-73.83%), 50.33% (95% CI: 9.935%-79.42%), 74.74% (95% CI: 25.21%-88.00%), and 59.16% (95% CI: 12.65%-83.56%) of baseline, respectively. The AUCs of the CDH3 response to chemotherapy was 0.900, and the serum CDH3 of distant metastatic CRCs in the PR group reduced to 38.8% (95% CI: 30.95%-53.77%) of baseline. Compared with serum tumor biomarkers CA24-2, CA19-9, CA72-4, and CEA in CRCs, CDH3 exhibited superior potential as a serum biomarker for monitoring chemotherapy response in distant metastatic CRCs. Our findings emphasize the potential of cadherin 3 (CDH3) as a dynamic biomarker for monitoring chemotherapy response. The observed correlation between CDH3 expression dynamics and treatment effect suggests that CDH3 can serve as a non-invasive tool for real-time evaluation of chemotherapy interventions, providing clinicians with a valuable resource for personalized treatment adjustments.

## Conclusion

Serum CDH3 is an effective serum biomarker for the diagnosis of distant metastatic CRCs and monitoring response to palliative chemotherapy in distant metastatic CRCs.

## Figures and Tables

**Table 1:** Clinical features of the study subjects.

Clinical features	NCs (n=28)	Non-metastasis (n=35)	Regional lymph node metastasis (n=37)	Distant metastasis (n=24)
Age (median (25th - 75th percentiles))	58.00 (45.75-65.00)	62.00 (52.00-66.00)	61.00 (54.00-68.50)	59.50 (52.50-65.50)
Gender (Male/Female)	13/15	22/13	30/7	14/10
Tumer size (> 2 cm)	/	35	35	24
Lymph node metastasis	/	0	37	24
Distant metastasis	/	/	/	24
Liver	/	/	/	18
Lung	/	/	/	1
Peritoneum and pelvis	/	/	/	2
Multiple metastases	/	/	/	3
Chemotherapy	/	/	/	24
XELOX	/	/	/	5
XELOX+bevacizumab	/	/	/	14
FOLFOX+bevacizumab	/	/	/	1
FOLFOX+cetuximab	/	/	/	1
FOLFIRINOX	/	/	/	1
FOLFIRINOX+bevacizumab	/	/	/	1
FOLFIRINOX+cetuximab	/	/	/	1

XELOX (capecitabine and oxaliplatin); FOLFOX (5-fluorouracil [5FU], folinic acid [LV], and oxaliplatin); FOLFIRINOX (5FU/LV, irinotecan and oxaliplatin)

**Table 2:** Relationship between the expression of CDH3, CA24-2, CA19-9, CA72-4, CEA and CRCs (Distant metastasis) [n (%)]

Feature	NCs and CRCs (Non-distant metastasis)	CRCs (Distant metastasis)	*X^2^*	*P*
Gender			0.293	0.588
Male	65 (52.4)	15 (12.1)		
Female	35 (28.2)	9 (7.2)		
Age (Year)			2.407	0.121
≤50	26 (21.0)	3 (2.4)		
>50	74 (59.7)	21 (16.9)		
CDH3 (ng/ml)			30.88	0.000
0-17.245	94 (75.8)	10 (8.1)		
>17.245	6 (4.8)	24 (19.4)		
CA24-2 (U/ml)			15.974	0.000
0-20	88 (71.0)	12 (9.7)		
>20	12 (9.7)	12 (9.7)		
CA19-9 (U/ml)			22.373	0.000
0-27	86 (69.4)	9 (7.3)		
>27	14 (11.3)	15 (12.1)		
CA72-4 (U/ml)			24.675	0.000
0-6.9	83 (66.9)	7(5.6)		
>6.9	17 (13.7)	17 (13.7)		
CEA (ug/ml)			14.407	0.000
0-5	67 (54.0)	6 (4.8)		
>5	33 (26.6)	18 (14.5)		

**Table 4:** Relationship between the expression of CDH3, CA24-2, CA19-9, CA72-4, CEA and CRCs (PR) [n (%)].

Tumor markers	Non-PR	PR	*X^2^*	*P*
CDH3 (ng/ml)			3.703	0.054
0-17.245	4 (16.7)	11 (45.8)		
>17.245	6 (25.0)	3 (12.5)		
CA24-2 (U/ml)			0.064	0.801
0-20	5 (20.8)	6 (25.0)		
>20	4 (16.7)	6 (25.0)		
CA19-9 (U/ml)			0.087	0.768
0-27	4 (16.7)	6 (25.0)		
>27	6 (25.0)	7 (29.2)		
CA72-4 (U/ml)			1.180	0.277
0-6.9	8 (33.3)	7 (29.1)		
>6.9	2 (8.3)	5 (20.8)		
CEA (ug/ml)			0.007	0.932
0-5	2 (8.3)	3 (12.5)		
>5	8 (33.3)	11 (45.8)		

**Table 5:** Relationship between the expression of CDH3, CA24-2, CA19-9, CA72-4, CEA (C3/C0) and CRCs (PR) [n (%)].

Tumor markers	Non-PR	PR	*X^2^*	*P*
CDH3 (C3/C0)			16.800	0.000
<1	2 (8.3)	14 (58.3)		
≥1	8 (33.3)	0 (0.0)		
CA24-2 (C3/C0)			0.875	0.350
<1	5 (20.8)	9 (37.5)		
≥1	4 (16.7)	3 (12.5)		
CA19-9 (C3/C0)			0.087	0.768
<1	5 (20.8)	10 (41.7)		
≥1	5 (20.8)	3 (12.5)		
CA72-4 (C3/C0)			1.721	0.190
<1	7 (29.2)	11 (45.8)		
≥1	3 (12.5)	1 (4.2)		
CEA (C3/C0)			0.137	0.711
<1	8 (33.3)	12 (50.0)		
≥1	2 (8.3)	2 (8.3)		

**Table 3:** Logistic regression analysis of Tumor markers expression and CRCs (Distant metastasis).

Tumor markers	*B*	*SE*	*Wald*	*P*	*OR*	*95% CI*
CDH3	3.551	0.951	13.955	0.000	34.852	5.408-224.58
CA24-2	0.119	1.223	0.01	0.922	1.127	0.102-12.392
CA19-9	2.509	1.118	5.037	0.025	12.298	1.374-110.046
CA72-4	2.133	0.766	7.757	0.005	8.437	1.881-37.841
CEA	0.039	0.926	0.002	0.967	1.039	0.169-6.389

## Abbreviations

AUC: the areas under the receiver operating characteristic curves
CA 19-9: carbohydrate antigen 19-9
CA 24-2: carbohydrate antigen 24-2
CA 72-4: carbohydrate antigen 72-4
CDH3: P-cadherin
CEA: carcinoembryonic antigen
CI: confidence interval
CR: complete response
CRC: colorectal cancer
CT: computed tomography
ELISA: enzyme-linked immunosorbent assay
EMT: epithelial-mesenchymal transition
HPA: Human Protein Atlas
IHC: immunohistochemistry
NC: normal control
PD: progressive disease
PR: partial response
RECIST: Response Evaluation Criteria In Solid Tumours
SD: stable disease
TCGA: The Cancer Genome Atlas
UGIA: upper gastrointestinal adenocarcinoma
